# Antioxidant Properties of Whey Protein‐Derived Peptides: Radical Scavenging and Cytoprotective Effects

**DOI:** 10.1002/fsn3.71092

**Published:** 2025-10-17

**Authors:** Ryota Suzuki, Sayuri Arai, Masaki Kurimoto, Naoki Yuda, Miyuki Tanaka

**Affiliations:** ^1^ Food Function Research Institute Morinaga Milk Industry Co., Ltd. Kanagawa Japan

**Keywords:** antioxidants, cytoprotective activity, peptide, radical scavenging activity, whey protein hydrolysate

## Abstract

Whey protein hydrolysates (WPH) are rich in peptides with potential antioxidant properties, yet the specific contributions of individual sequences remain underexplored. In this study, we identified eight antioxidant peptides—GYDTQ, GY, VY, DTDYKKY, WYS, WY, LDQW, and YW—from WPH by isolating fractions with high hydrophilic‐oxygen radical absorbance capacity (H‐ORAC) activity. Among them, GYDTQ was identified as a novel antioxidant peptide. The antioxidant activities of these peptides were further evaluated using 2, 2‐diphenyl‐1‐picrylhydrazyl (DPPH) and 2, 2‐azino‐bis(3‐ethylbenzothiazoline‐6‐sulfonic acid) (ABTS) radical scavenging assays. All eight peptides exhibited radical scavenging activity: LDQW showed the highest H‐ORAC value (3.79 ± 0.15 μmol Trolox equivalents [TE]/μmol), YW demonstrated the strongest DPPH activity (22.45% ± 1.75%), and WYS exhibited the highest ABTS activity (1.29 ± 0.06 mM TE/mM peptide). Cytoprotective assays using murine C2C12 myoblast cells revealed that DTDYKKY, GYDTQ, LDQW, and WY significantly restored cell viability under hydrogen peroxide‐induced oxidative stress, with DTDYKKY showing the greatest effect. Notably, GYDTQ exhibited low radical scavenging activity but high cytoprotective potential, suggesting alternative antioxidant mechanisms. These results indicate that WPH and its constituent peptides represent potential functional foods to prevent the symptoms of oxidative stress and exercise‐induced fatigue.

## Graphical abstract

In this study, whey protein hydrolysate was fractionated and several antioxidant peptides were identified from the fraction with high H‐ORAC activity. These peptides exhibited H‐ORAC, DPPH, and ABTS radical scavenging activity and cytoprotective effects in C2C12 cells.

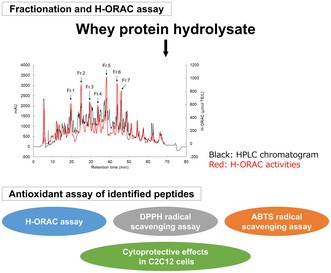

## 1 Introduction

Oxidative stress damages cells and tissues, leading to the development of conditions such as aging, cancer, and cardiovascular disease (Dubois‐Deruy et al. 2020). Foods with antioxidant capacities can mitigate oxidative stress in animals and humans, potentially improving fatigue and preventing aging (Lee, Hsu, et al. 2021; Wang et al. 2012). Therefore, antioxidants play an important role in maintaining health.

Antioxidant peptides exert their effects by interacting with free radicals and are expected to contribute to the prevention and treatment of various diseases caused by oxidative stress (He et al. 2019; Wang et al. 2025). Antioxidant peptides have been identified in multiple foods, including fish, eggs, soybeans, grains, and milk (Sarmadi and Ismail 2010). The Milk Bioactive Peptide Database summarizes information on milk‐derived bioactive peptides, including antioxidants, that have been discovered to date (Nielsen et al. 2024). Although the antioxidant activity of milk protein hydrolysates has been reported (Rival et al. 2001; Xie et al. 2013), the specific properties of individual peptides remain underexplored.

Several radical scavenging assays have been developed to assess antioxidant capacity. The radical scavenging assays, such as the ORAC assay, DPPH radical scavenging assay, and ABTS radical scavenging assay, are commonly used to evaluate the antioxidant activity of foods. These assays differ in radical species and mechanisms, which can influence the observed activity depending on assay conditions (Huang et al. 2002; Shimamura et al. 2014; Tang et al. 2010; Thaipong et al. 2006; Zheng et al. 2015). Therefore, comparing the activity obtained from multiple radical scavenging methods is necessary.

In addition to performing radical scavenging assays, the antioxidant capacity should be evaluated in cultured cells or in vivo. The C2C12 cell line isolated from muscular dystrophic mice is widely used to study muscle differentiation and oxidative stress (Yaffe and Saxel 1977). Food‐derived compounds, such as curcumin and fisetin, have been shown to restore the viability of C2C12 myoblasts following hydrogen peroxide (H_2_O_2_)‐induced oxidative damage, suggesting their protective effects against muscle injury (Lee, Chun, et al. 2021; Park et al. 2023). Therefore, identifying peptides capable of restoring C2C12 cell viability under oxidative stress conditions may contribute to the development of functional ingredients for the prevention of muscle atrophy and movement disorders (Jackson and O'Farrell 1993; Pansarasa et al. 1999).

This study aimed to identify potential antioxidant peptides in whey protein hydrolysate (WPH) by comparing multiple antioxidant activity assays in vitro, including cell‐based assays. We identified eight antioxidant peptides with radical scavenging activity and cytoprotective effects against oxidative stress. The high antioxidant activity of the peptides identified in this study suggests their potential utility in the development of peptide‐based therapeutics targeting oxidative stress‐related conditions, such as neurodegenerative and cardiovascular diseases, as well as in the formulation of functional foods and dietary supplements aimed at fatigue reduction and aging prevention.

## 2 Materials and Methods

### 2.1 Materials and Reagents

WPH (product name WPH‐M), which had a nutritional composition of 78.2% protein, 0.4% fat, 12.3% carbohydrate, 5.1% ash, and 4.0% moisture, was obtained from Morinaga Milk Industry (Tokyo, Japan). It was enzymatically digested from whey protein concentrate (WPC), with a degree of hydrolysis of 20.5% determined by the formol titration method and an average molecular weight of 448 Da determined by gel permeation chromatography. The amino acid composition was determined by mass spectrometry and is shown in Table S1.

Dulbecco's modified Eagle's medium (DMEM, cat# 043–30,085), penicillin–streptomycin solution (cat# 168–23,191), trifluoroacetic acid (TFA, cat# 204–10,771), formic acid (FA, cat# 067–04531), acetic acid (cat# 017–00256), reduced‐form glutathione (GSH, cat# 073–02013), 30% H_2_O_2_ solution (cat# 080–01186), ethanol (cat# 057–00456), and methanol (cat# 131–01826) were purchased from FUJIFILM Wako Pure Chemical (Tokyo, Japan). Fetal bovine serum (FBS, cat# 26140079) was purchased from Thermo Fisher Scientific (Waltham, MA, USA). High‐performance liquid chromatography (HPLC)‐grade water (cat# 11,307–1B), acetonitrile (cat# 01033–79), and methanol (cat# 25,183–1B) were purchased from Kanto Chemical (Tokyo, Japan). Cadenza CD C18 HPLC columns (10 × 250 mm, cat# CD0P6) were purchased from Imtakt (Kyoto, Japan), and ACQUITY UPLC BEH C18 columns (2.1 × 150 mm, cat# 186002353) were purchased from Waters (Milford, MA, USA). Peptides with sequences of GYDTQ, GY, VY, DTDYKKY, VL, WYS, WY, LDQW, and YW were purchased from BEX (Tokyo, Japan), and their purities exceeded 90%.

### 2.2 Fractionation of Peptides With High H‐ORAC Activity From WPH

The WPH was fractionated using HPLC (UltiMate 3000; Thermo Fisher Scientific, Waltham, MA, USA). The WPH was dissolved in ultrapure water to a concentration of 10 mg/mL and then filtered through a 0.22‐*μm* membrane (Millex‐GV; cat# SLGVR33RS, Merck, Darmstadt, Germany). Subsequently, it was placed in a Cadenza CD C18 HPLC column (10 × 250 mm). Samples (2 mL) were eluted at a flow rate of 3 mL/min for 80 min using a gradient of 0.1% TFA in water (solvent A) and 0.1% TFA in acetonitrile (solvent B) as follows: 2%–30% B for 60 min, 30%–80% B for 10 min, and 2% B for 10 min. Elution was monitored at a wavelength of 215 nm. In total, 192 fractions were collected every 20 s from 5 to 69 min, concentrated, and dried at 40°C using a vacuum centrifugal evaporator (MiVac Quattro Concentrator; Biopharma Process Systems, Winchester, United Kingdom). Subsequently, the dried samples were diluted in 10% acetonitrile and further analyzed via the hydrophilic ORAC (H‐ORAC) assay, as described below.

Fractions with high H‐ORAC activity were further fractionated using HPLC. These fractions were redissolved in a 0.1% TFA solution and loaded onto a Cadenza CD C18 column. Samples (2 mL) were fractionated using a gradient of 0.1% TFA in water (solvent A) and 0.1% TFA in acetonitrile (solvent B) with the flow rates and gradients specifically adjusted for each fraction (Table S2). Elution was monitored at a wavelength of 215 nm. H‐ORAC assays were performed again based on the major peaks observed in the chromatogram, and a fraction with high H‐ORAC activity was obtained (Figure S1).

### 2.3 Peptide Identification Using Liquid Chromatograph–Mass Spectrometry

Upon repeated fractionation, fractions that exhibited high H‐ORAC activity were subjected to liquid chromatography (LC) (Vanquish UHPLC; Thermo Fisher Scientific, Waltham, MA, USA) for peptide separation. The active fraction was reconstituted in ultrapure water and loaded onto the ACQUITY UPLC BEH C18 column (2.1 × 150 mm). An aliquot of the 10 μL sample was eluted using a gradient of 0.1% FA in water (solvent A) and 0.1% FA in acetonitrile (solvent B) at a flow rate of 0.2 mL/min for 45 min. The gradient program was as follows: 2%–30% B for 30 min, 30%–90% B for 5 min, 90%–2% B for 1 min, and held at 2% B for 9 min.

The mass of the peptides present in each fraction was determined using a Fourier transform mass spectrometer (Q Exactive Focus; Thermo Fisher Scientific, Waltham, MA, USA), and each amino acid sequence was estimated by comparing its mass with that of the whey protein‐derived peptide sequence. The amino acid sequences of *α*‐lactalbumin (*α*‐LA) and *β*‐lactoglobulin (*β*‐LG), the main proteins in whey, were retrieved from the NCBI protein database (https://www.ncbi.nlm.nih.gov/protein/), and the theoretical molecular weights of peptide fragments generated by enzymatic digestion were calculated and compared with those of the observed full‐mass spectrometry (MS). Peptides were identified by comparison with corresponding synthetic peptide standards using LC‐tandem MS (MS/MS).

Full‐MS scans were performed within the *m/z* range of 120–1800. The parameters for tandem MS measurements were as follows: MS resolution at 70,000, MS/MS resolution at 17,500, sheath gas flow rate at 45 units, auxiliary gas flow rate at 10 units, spray voltage at 3.5 kV, capillary temperature at 275°C, and auxiliary gas heater temperature at 350°C.

### 2.4 H‐ORAC Assay

The assay was conducted using the H‐ORAC activity assay kit (cat# 295–79,501, FUJIFILM Wako Pure Chemical, Osaka, Japan), referring to Watanabe et al. (2012). Briefly, the samples were diluted in a 10% methanol/water/acetic acid solution and added to the wells along with Trolox calibration solutions (6.25, 12.5, 25, and 50 μM) or blanks (35 μL). Next, fluorescein (115 μL) and 2,2′‐azobis (2‐methylpropionamidine) dihydrochloride (50 μL) were added. The solutions were incubated in a 96‐well plate (Multi Well Plate for Suspension Culture 96F with lid; cat# MS‐8096R, Sumitomo Bakelite, Tokyo, Japan) and sealed with plastic film at 37°C. Fluorescence (excitation, 485 nm; emission, 530 nm) was monitored every 2 min for 90 min using a multiplate reader (SH‐9000Lab; Corona Electric, Hitachinaka, Japan). The net area under the curve (AUC) was calculated by subtracting the blank AUC from the AUC of the sample or standard. The H‐ORAC value for each sample was determined by comparing the net AUC with that of the Trolox standard. The units are expressed in μmol trolox equivalent (TE)/g or μmol TE/μmol.

### 2.5 DPPH Radical Scavenging Assay

Similar to previous studies that evaluated the antioxidant activity of peptides (Wang, Li, et al. 2022), the DPPH radical scavenging capacity of the samples was assessed. The assay was conducted using the DPPH Antioxidant Assay Kit (cat# D678, Dojindo, Kumamoto, Japan). Briefly, 20 μL of the sample was added to each well, followed by the addition of 80 μL of assay buffer. Subsequently, 100 μL of DPPH working solution was added, and the mixture was incubated for 30 min in the dark at room temperature (25°C). The absorbance was measured at 517 nm using a multiplate reader, and the results are expressed as a percentage of radical scavenging activity. The absorbance of the control solution (A_CS_) was calculated by subtracting the absorbance of the solution containing the solvent instead of the sample from that of the solution containing ethanol instead of the DPPH working solution. Similarly, the absorbance of the sample (A_S_) was calculated by subtracting its absorbance from that of the solution containing ethanol instead of the DPPH working solution.

$$\text{Radical scavenging rate}\;\left(\%\right)=\left(A_{\mathrm{CS}}–A_{S}\right)/A_{\mathrm{CS}}\times 100$$

### 2.6 ABTS Radical Scavenging Assay

The assay was conducted using the antioxidant assay kit (cat# 709001, Cayman Chemical, Ann Arbor, MI, USA) as previously described (Bomble and Nath 2022). Briefly, 10 μL of either the sample or Trolox calibration solution (30, 60, 90, 120, 150, and 220 μM) was dispensed into the wells. This was followed by the sequential addition of 10 μL of metmyoglobin solution and 150 μL of chromogen solution. The reaction was initiated by the addition of 40 μL of a 441 μM H_2_O_2_ working solution. The plate was sealed with a plastic film and allowed to react for 5 min at room temperature. The absorbance was measured at 405 nm using a multiplate reader. The net absorbance was calculated by subtracting the absorbance of the blank and is expressed in mM TE/mM peptide as Trolox equivalents.

### 2.7 Cell Culture

The C2C12 murine myoblast cell line (cat# 91031101) was procured from The European Collection of Authenticated Cell Cultures (Salisbury, United Kingdom). The cells were maintained in DMEM containing 10% FBS and 1% penicillin/streptomycin. Subsequently, the cells were incubated at 37°C in a humidified atmosphere containing 5% CO_2_. Upon reaching approximately 80% confluence, the cells were passaged onto fresh culture dishes.

### 2.8 Cell Viability Assay

Cell viability was evaluated in C2C12 myoblasts according to the procedure reported by Lee et al. (2013), with slight modifications to suit the experimental conditions. C2C12 myoblasts were seeded into 96‐well plates at a density of 1 × 10^4^ cells/well and incubated for 24 h. After incubation, the cells were rinsed with DMEM without FBS and penicillin/streptomycin. Subsequently, samples were added to the culture, and the cells were incubated for an additional 24 h. Next, varying concentrations of H_2_O_2_ were added, and the cells were incubated for 1 h. The cells were subsequently rinsed again, and cell viability was assessed using Cell Counting Kit‐8 (CCK‐8; cat# CK04, Dojindo, Kumamoto, Japan). Specifically, a culture medium was added to the rinsed wells, followed by the addition of 1% CCK‐8 solution. After incubation for 2 h, the absorbance was measured at 450 nm using a multiplate reader. The cell viability was determined using the following formula:

$$\text{Cell viability}\;\left[\%\right]=\left(\text{sample}\;\mathrm{OD}/\text{control}\;\mathrm{OD}\right)\times 100$$

where OD refers to optical density.

### 2.9 Statistical Analysis

To determine the significance of differences between the control and treatment groups, data were analyzed using Student's *t*‐test or one‐way analysis of variance, followed by Dunnett's test. Data are presented as the mean ± standard deviation. All statistical analyses were performed using SPSS Statistics version 29.0 (IBM, Armonk, NY, USA).

## 3 Results

### 3.1 Evaluation of H‐ORAC Activity in WPC and WPH

**FIGURE 1:**
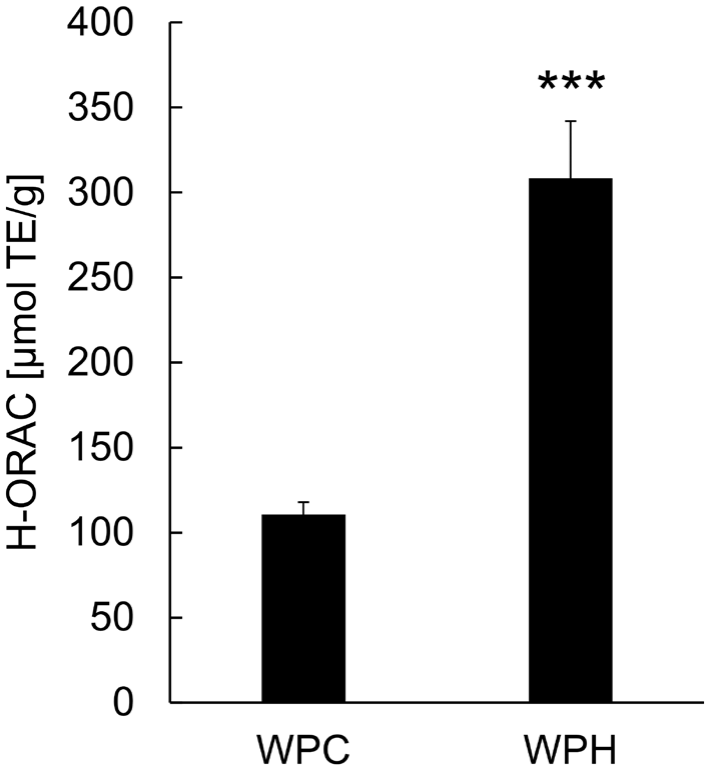
H‐ORAC activity of WPH. The H‐ORAC activity of WPH was measured using WPC as a control. The results are expressed as the mean ± standard deviation of three independent experiments. Statistical significance was determined using Student's *t*‐test. Asterisks (***) indicate *p* < 0.001 compared to the result of the WPC. H‐ORAC, hydrophilic‐oxygen radical absorbance capacity; TE, Trolox equivalents; WPH, whey protein hydrolysate; WPC, whey protein concentrate.

The H‐ORAC assay was conducted to evaluate the antioxidant activities of the samples. WPC exhibited an H‐ORAC value of 110.74 ± 7.17 μmol TE/g, whereas that of WPH was higher at 308.22 ± 33.82 μmol TE/g (Figure 1). This suggests that WPH possessed greater antioxidant activity than WPC, owing to the antioxidant properties of the peptides in WPH.

### 3.2 Evaluation of H‐ORAC Activity in Peptides of WPH

**FIGURE 2:**
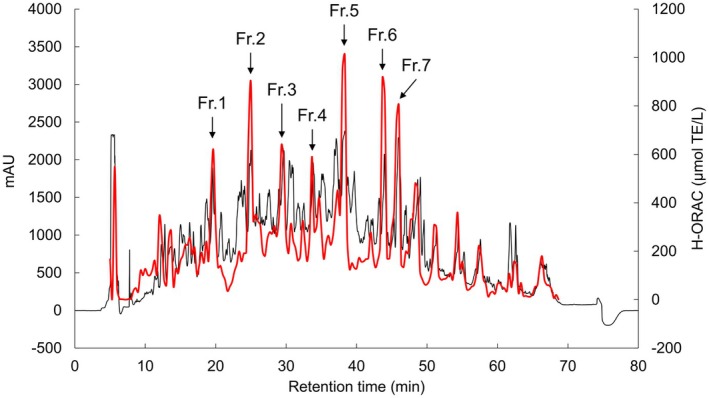
HPLC chromatogram and H‐ORAC activity of the WPH. WPH was fractionated using HPLC, and the H‐ORAC activity of each fraction was measured. The HPLC chromatogram is shown in black and the H‐ORAC values of the fractions are shown in red. Arrows indicate the fractions with high H‐ORAC activities, which are labeled in the order of their retention times as Fr.1–Fr.7. H‐ORAC, hydrogen‐oxygen radical absorbance capacity; HPLC, high‐performance liquid chromatography; WPH, whey protein hydrolysate.

We identified the antioxidant peptides in WPH that exhibited higher H‐ORAC values than those in WPC. WPH was fractionated using hydrophobic interaction HPLC, and the H‐ORAC values were determined for 192 fractions collected between 5 and 69 min (Figure 2). Seven fractions (designated as Fr.1–Fr.7 in order of increasing retention time) had markedly high H‐ORAC values. Each of these seven fractions was further fractionated using HPLC, and fractions with high H‐ORAC activity were obtained (Figure S1). For each fraction, the most dominant fragment ion was identified using LC–MS (Figure S2) and subjected to MS/MS. As described in Table 1, the following antioxidant peptide candidates were identified from the major fragment ions in Fr.1–Fr.7: GYDTQ and GY from Fr.1; VY from Fr.2; DTDYKKY from Fr.3; VL from Fr.4; WYS from Fr.5; WY from Fr.6; and LDQW and YW from Fr.7.

**TABLE 1:** Identification of peptide candidates in whey protein hydrolysate.

HPLC fraction	*m/z*	*z*	Estimated molecular mass (Da)	Sequence
Fr.1–1	583.23	1	582.23	GYDTQ
Fr.1–2	239.10	1	238.09	GY
Fr.2–1	281.15	1	280.14	VY
Fr.3–1	311.48	3	931.42	DTDYKKY
Fr.4–1	231.17	1	230.16	VL
Fr.5–1	455.19	1	454.18	WYS
Fr.6–1	368.16	1	367.15	WY
Fr.7–1	561.26	1	560.26	LDQW
Fr.7–2	368.16	1	367.15	YW

Abbreviation: HPLC, high‐performance liquid chromatography.

### 3.3 H‐ORAC Assay of Peptides in WPH

**FIGURE 3:**
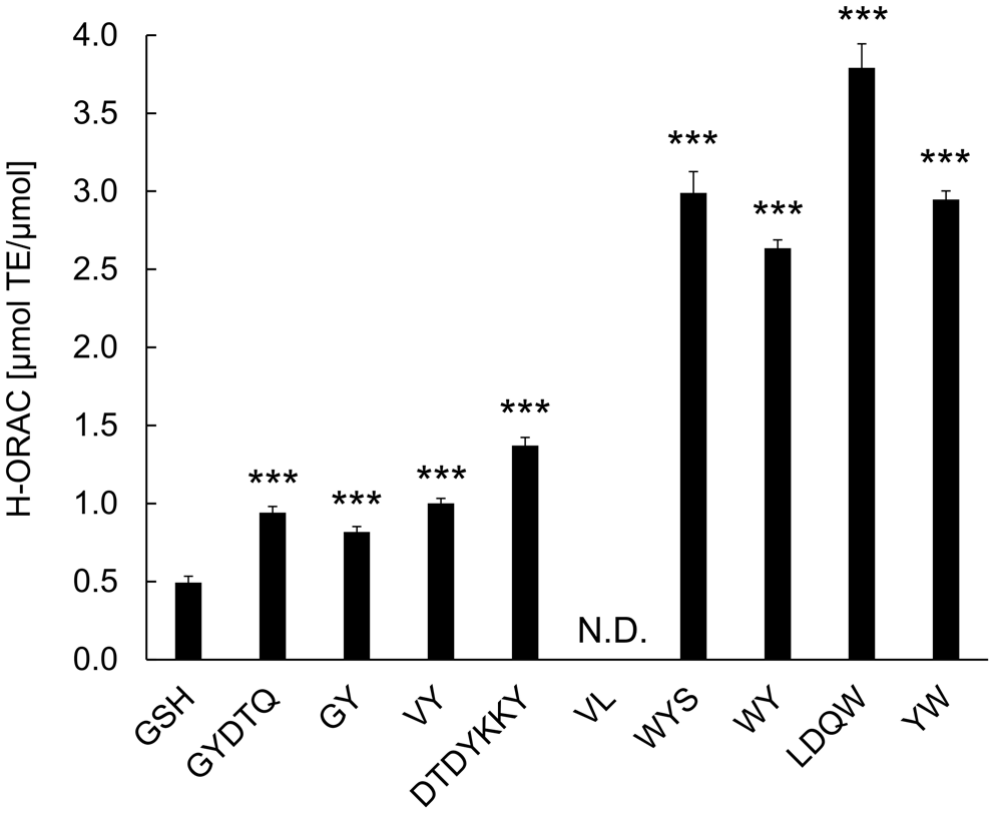
H‐ORAC activity of peptides in the WPH. The antioxidant activity of peptides in the WPH was measured using the H‐ORAC assay. GSH was used as a control. The results are expressed as the mean ± standard deviation of four independent experiments. Statistical analysis was performed using one‐way analysis of variance followed by Dunnett's test to compare each peptide with GSH. Peptides that showed significantly higher antioxidant activity than GSH are indicated using asterisks (***p < 0.001). GSH, glutathione; H‐ORAC, hydrogen‐oxygen radical absorbance capacity; N.D., not detected; TE, trolox equivalents; WPH, whey protein hydrolysate.

The antioxidant activities of the peptides in WPH were determined using the H‐ORAC assay. The eight peptides GYDTQ (0.94 ± 0.04 μmol TE/μmol), GY (0.82 ± 0.03 μmol TE/μmol), VY (1.00 ± 0.03 μmol TE/μmol), DTDYKKY (1.37 ± 0.05 μmol TE/μmol), WYS (2.99 ± 0.14 μmol TE/μmol), WY (2.63 ± 0.05 μmol TE/μmol), LDQW (3.79 ± 0.15 μmol TE/μmol), and YW (2.95 ± 0.06 μmol TE/μmol) showed greater H‐ORAC activity than GSH (0.49 ± 0.15 μmol TE/μmol) (Figure 3). Among the eight peptides, LDQW showed the highest H‐ORAC value. Peptide VL did not exhibit H‐ORAC activity.

### 3.4 DPPH and ABTS Radical Scavenging Assays of WPH Peptides

For the eight peptides that demonstrated high H‐ORAC activity, additional radical scavenging assays, including DPPH and ABTS radical scavenging assays, were conducted (Table 2). In the DPPH radical scavenging assay, the peptide YW exhibited the highest activity, with a radical scavenging rate of 22.45% ± 1.75%, followed by WY (17.75% ± 1.32%), LDQW (13.19% ± 1.06%), WYS (12.65% ± 1.36%), DTDYKKY (9.65% ± 2.15%), VY (2.01% ± 1.93%), GY (2.01% ± 0.88%), and GYDTQ (0.69% ± 1.62%). In the ABTS radical scavenging assay, the peptide WYS exhibited the highest activity at 1.29 ± 0.06 mM TE/mM peptide, followed by DTDYKKY (0.88 ± 0.08 mM TE/mM peptide), WY (0.44 ± 0.14 mM TE/mM peptide), YW (0.41 ± 0.07 mM TE/mM peptide), GY (0.40 ± 0.10 mM TE/mM peptide), VY (0.31 ± 0.07 mM TE/mM peptide), LDQW (0.23 ± 0.02 mM TE/mM peptide), and GYDTQ (0.06 ± 0.01 mM TE/mM peptide). All eight peptides that exhibited radical scavenging activity in the H‐ORAC assay also demonstrated activity in both the DPPH and ABTS assays. A similar trend was observed between the H‐ORAC and DPPH radical scavenging assay results, with the top five peptides (DTDYKKY, WYS, WY, LDQW, and YW) showing the highest activity in both assays. The ABTS radical scavenging assay results showed a different trend from the H‐ORAC and DPPH radical scavenging assay results. In ABTS radical scavenging assays, WYS and DTDYKKY exhibited relatively higher activity than the other peptides. Additionally, GY, which showed low values in the H‐ORAC and DPPH radical scavenging assays, was as active as WY and YW, which showed higher values in the H‐ORAC and DPPH radical scavenging assays.

**TABLE 2:** DPPH and ABTS activities of peptides in whey protein hydrolysate.

Sequence	DPPH radical scavenging activity (Radical scavenging rate [%])	ABTS radical scavenging activity (mM TE/mM peptide)
GYDTQ	0.69 ± 1.62	0.06 ± 0.01
GY	2.01 ± 0.88	0.40 ± 0.10
VY	2.01 ± 1.93	0.31 ± 0.07
DTDYKKY	9.65 ± 2.15	0.88 ± 0.08
WYS	12.65 ± 1.36	1.29 ± 0.06
WY	17.75 ± 1.32	0.44 ± 0.14
LDQW	13.19 ± 1.06	0.23 ± 0.02
YW	22.45 ± 1.75	0.41 ± 0.07

Abbreviations: ABTS, 2,2′‐azino‐bis(3‐ethylbenzothiazoline‐6‐sulfonic acid); DPPH, 2,2‐diphenyl‐1‐picrylhydrazyl; TE, Trolox equivalent.

### 3.5 Evaluation of the Cytoprotective Effects of WPH‐Derived Peptides Against Oxidative Stress

**FIGURE 4:**
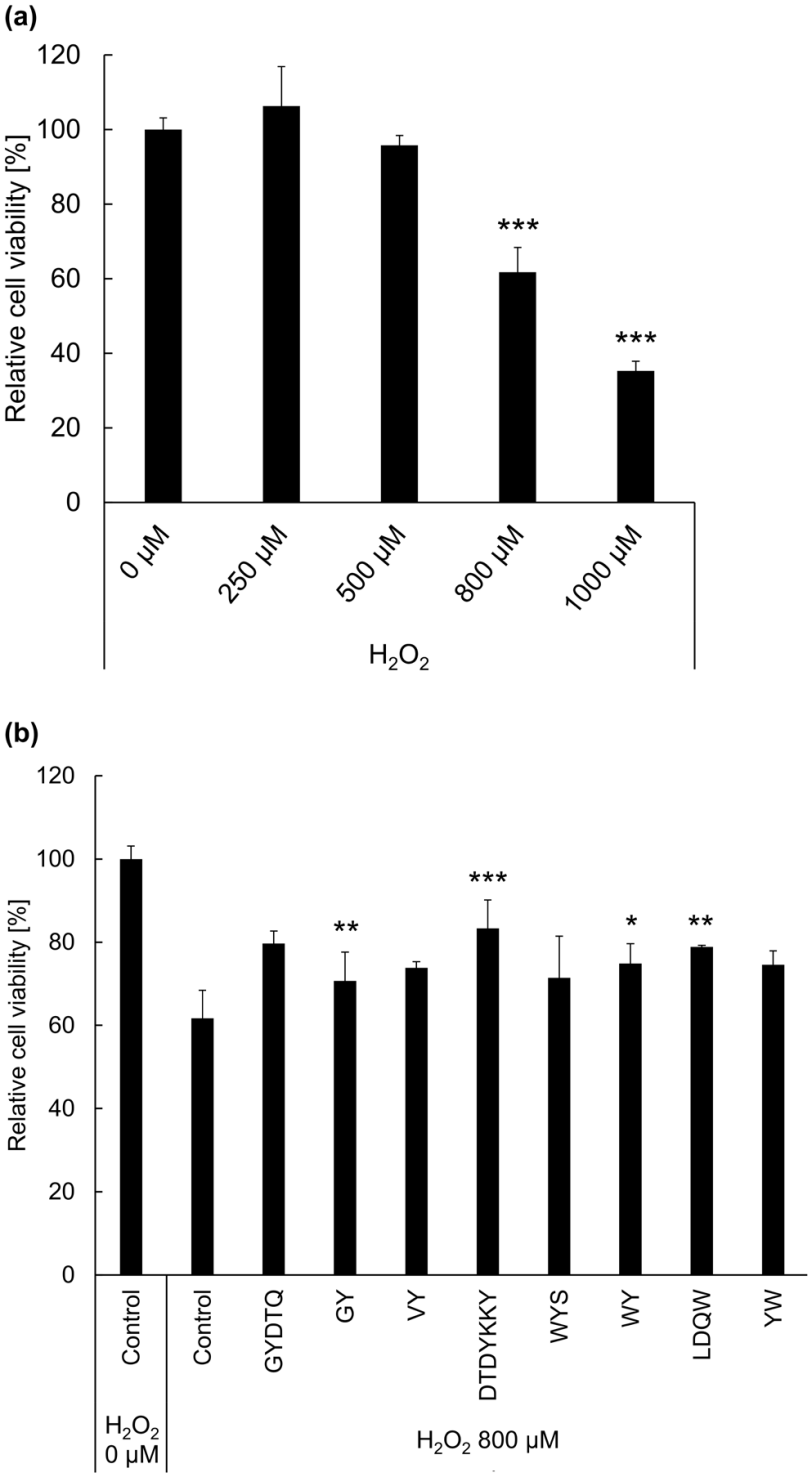
Effect of antioxidant peptides on C2C12 viability under oxidative stress induced by H_2_O_2_ treatment. Cell viability was assessed using a CCK‐8 assay. (a) C2C12 myoblasts were treated with varying concentrations of H_2_O_2_ (0–1000 μM) for 1 h. (b) C2C12 myoblasts were treated with 5 μM peptides that demonstrated radical scavenging activity. Following peptide treatment for 24 h, the cells were exposed to 800 μM H_2_O_2_ for 1 h to induce oxidative stress. Data are presented as the mean ± standard deviation from three independent experiments. Statistical analysis was performed using one‐way analysis of variance followed by Dunnett's test to compare (a) each dose of H_2_O_2_ treatment with the control (0 μM H_2_O_2_) or (b) each peptide with the control (800 μM H_2_O_2_). Peptides that significantly increased cell viability compared to that of the control are indicated by asterisks (***p < 0.001, **p < 0.01, and *p < 0.05). H_2_O_2_, hydrogen peroxide.

We investigated the effects of eight WPH‐derived peptides with recognized radical scavenging activities on the viability of C2C12 myoblasts. Initially, we assessed the effect of H_2_O_2_ on C2C12 cells across a concentration range up to 1000 μM. Cell viability was significantly reduced at concentrations of 800 and 1000 μM (Figure 4a). Dose–response experiments demonstrated that treatment with 800 μM H_2_O_2_ resulted in approximately 60% cell viability. This viability level aligns with previous studies (Jeong et al. 2022; Park et al. 2021), and thus, 800 μM was adopted as the optimal concentration of H_2_O_2_ for inducing oxidative stress in the present study. In C2C12 myoblasts subjected to H_2_O_2_‐induced oxidative stress, GYDTQ, DTDYKKY, WY, and LDQW significantly increased the cell viability compared with the control, with DTDYKKY having the highest viability restoration effect (Figure 4b). Even peptides with lower radical scavenging activities, including the novel peptide GYDTQ, increased cell viability. This suggests that these peptides exert their protective effects via mechanisms other than radical scavenging activity.

## 4 Discussion

In this study, we identified several antioxidant peptides of WPH by performing various radical scavenging assays and evaluating changes in the viability of C2C12 myoblasts. WPH was found to have a higher H‐ORAC value than WPC. The fraction with the highest H‐ORAC activity was identified using HPLC, and the antioxidant peptides of WPH were identified via LC–MS/MS. These peptides were active in the DPPH and ABTS radical scavenging assays, in addition to the H‐ORAC assay, and showed cytoprotective effects. Although there were several known sequences among them, a novel sequence, GYDTQ, was discovered.

The antioxidant properties of peptides are primarily attributed to their amino acid sequences. Tyrosine (Tyr), tryptophan (Trp), histidine, methionine, cysteine, and lysine are important determinants of the antioxidant capacity of bioactive peptides (Kumar et al. 2017; Ohashi et al. 2015). The peptides obtained in this study must contain Tyr or Trp residues, as the H‐ORAC assay detects the antioxidant activity of Tyr and Trp (Li and Li 2013). Among the identified peptide candidates, VL showed no H‐ORAC activity. In LC–MS/MS analysis of Fr.4 that contained VL, several peaks were observed in addition to the peak for VL, suggesting that the activity was due to a mixture of sequences containing amino acids with high antioxidant capacity, including Tyr or Trp.

The results of three radical scavenging assays (H‐ORAC, DPPH, and ABTS) were compared in this study. For the eight identified antioxidant peptides, the H‐ORAC and DPPH radical scavenging assays showed similar trends, whereas the ABTS radical scavenging assay showed a different trend. Although the DPPH and ABTS radical scavenging assays are similar evaluation methods, the radical scavenging activity depends on the polarity of antioxidants, with DPPH reacting to hydrophobic substances but ABTS reacting to both hydrophilic and lipophilic substances (Rumpf et al. 2023; Tang et al. 2010). The ABTS radical scavenging assay is characterized by a short reaction time, and previous studies have reported that reaction speed may vary depending on the sample (Dong et al. 2015). Accordingly, WYS and DTDYKKY, which showed relatively high ABTS radical scavenging activity, are considered to be peptides with rapid reactivity. We assumed that such peptide characteristics affected the differences between the assay results in this study. This also suggests the need to combine multiple radical scavenging assays rather than using a single method.

In this study, we identified eight antioxidant peptides (Table 3). The peptide GYDTQ has been newly identified, and there is currently no evidence supporting its antioxidant activity or any other biological functions. GYDTQ showed the lowest antioxidant activity among the eight antioxidant peptides in the three radical scavenging assays; however, it provided the second‐highest oxidative stress protection in C2C12 cells. This suggests that GYDTQ exerts its antioxidant effect via another mechanism, in addition to radical scavenging. Along with the novel antioxidant peptide GYDTQ, this study is the first to demonstrate that DTDYKKY and LDQW can protect cells under oxidative stress conditions. Notably, the three peptides showing the strongest cytoprotective effects—DTDYKKY, GYDTQ, and LDQW—share a common structural feature: the presence of aspartic acid residues. One example of an intracellular antioxidant mechanism is the activation of the Kelch‐like ECH‐associated protein 1 (Keap1)‐NF‐E2‐related factor‐2 (Nrf2) signaling pathway. The Kelch domain of Keap1 contains positively charged residues, such as arginine, which interact electrostatically with the negatively charged regions of Nrf2. Previous studies have shown that antioxidant peptides can bind to Keap1, thereby inhibiting its binding to Nrf2 and exerting antioxidant effects (Li et al. 2025; Zhu et al. 2022). This interaction increases the activity of intracellular antioxidant enzymes (glutathione peroxidase, superoxide dismutase, and catalase) (Wang, Liu, et al. 2022). Given that DTDYKKY, GYDTQ, and LDQW—three peptides exhibiting high cytoprotective effects—contain aspartic acid residues, it is plausible that these sequences may engage in electrostatic interactions with the positively charged Kelch domain of Keap1 and contribute to their observed protective effects under oxidative stress. In addition to the Nrf2‐Keap1 pathway, other signaling cascades—including the mitochondria‐dependent apoptosis pathway, transforming growth factor‐beta (TGF‐*β*)/SMAD, AMP‐activated protein kinase (AMPK)/sirtuin 1 (SIRT1)/peroxisome proliferator‐activated receptor gamma coactivator 1‐alpha (PGC‐1*α*), phosphoinositide 3‐kinase (PI3K)/protein kinase B (Akt)/mechanistic target of rapamycin (mTOR), and nuclear factor kappa‐light‐chain‐enhancer of activated B cells (NF‐κB) pathway—may also be implicated (Zhang et al. 2024). Further studies are required to determine the effects of antioxidant peptides on myoblasts.

**TABLE 3:** References for the antioxidant peptides identified in this study.

Sequence	Parental protein	References
GYDTQ	*α*‐La	This study
GY	*α*‐La	Ozturk‐Kerimoglu et al. (2023); Jiang et al. (2021)
VY	*β*‐Lg	Torkova et al. (2015); Du et al. (2020); Wu et al. (2023); Wu et al. (2024)
DTDYKKY	*β*‐Lg	Zhang et al. (2020)
WYS	*β*‐Lg	Hernández‐Ledesma et al. (2007); Nongonierma and Fitzgerald (2015); Zhu et al. (2020)
WY	*β*‐Lg	Torkova et al. (2015); Hernández‐Ledesma et al. (2007); Nongonierma and Fitzgerald (2015); Shi et al. (2022)
LDQW	*α*‐La	Sadat et al. (2011)
YW	*α*‐La	Torkova et al. (2015)

Abbreviations: *α*‐La, *α*‐lactalbumin; *β*‐Lg, *β*‐lactogloblin.

In conclusion, we showed the radical scavenging and protective effects of peptides derived from WPH on C2C12 cells. Among them, we identified a novel GYDTQ peptide. As oxidative stress is a cause of a variety of conditions, including aging, cardiovascular disease, and depression, the potential scope of antioxidant applications is significant. Although further research is needed to determine the precise effects of WPH and its identified peptides in animal models and human cohorts, the present findings support their potential as effective antioxidant agents. The peptides identified in this study suggest potential applications in the development of peptide‐based therapeutic agents for oxidative stress‐related diseases, as well as in the formulation of functional foods and supplements containing food protein hydrolysates rich in these peptides for alleviation of fatigue and anti‐aging purposes.

## Author Contributions

**Ryota Suzuki:** conceptualization (equal), data curation (lead), formal analysis (lead), investigation (lead), methodology (lead), validation (lead), visualization (lead), writing – original draft (lead), writing – review and editing (equal). **Sayuri Arai:** conceptualization (equal), data curation (equal), methodology (equal), validation (equal), visualization (equal), writing – review and editing (equal). **Masaki Kurimoto:** conceptualization (equal), data curation (equal), methodology (equal), project administration (equal), resources (equal), validation (equal), visualization (equal), writing – review and editing (equal). **Naoki Yuda:** conceptualization (lead), funding acquisition (equal), methodology (equal), project administration (lead), resources (lead), validation (equal), visualization (equal), writing – review and editing (lead). **Miyuki Tanaka:** conceptualization (equal), funding acquisition (lead), supervision (lead), writing – review and editing (equal).

## Ethics Statement

This study does not involve any human or animal testing.

## Conflicts of Interest

This work was supported by the Morinaga Milk Industry Co. Ltd., Tokyo, Japan. R.S., S.A., M.K., N.Y., and M.T. are employed by the Morinaga Milk Industry Co. Ltd., Tokyo, Japan. There are no other conflicts of interest.

## Supporting information


**Table S1:** Amino acid composition of the whey protein hydrolysate.
**Table S2:** Fractionation conditions for each fraction.
**Figure S1:** HPLC chromatogram and H‐ORAC activity of each fraction. Frs.1–7, which had high H‐ORAC activity among the 192 fractions of whey protein hydrolysate, were fractionated again, and H‐ORAC activity was measured. The HPLC chromatogram is shown in black, and the H‐ORAC values of the fractions are indicated in red. Arrows indicate fractions with high H‐ORAC activity. Graphs show: (A) Fr.1, (B) Fr.2, (C) Fr.3, (D) Fr.4, (E) Fr.5, (F) Fr.6, and (G) Fr.7. H‐ORAC; hydrogen‐oxygen radical absorbance capacity; HPLC, high‐performance liquid chromatography; TE, Trolox equivalent.
**Figure S2:** Dominant fragment ions of each fraction. Each fraction was refractionated and subjected to LC–MS/MS to determine the fraction with the highest hydrogen‐oxygen radical absorbance capacity activity. The figure shows the dominant fragments in LC–MS. The vertical axis presents the relative abundance, and the horizontal axis presents the *m/z* values. The respective graphs show: (A) Fr.1–1, (B) Fr.1–2, (C) Fr.2–1, (D) Fr.3–1, (E) Fr.4–1, (F) Fr.5–1, (G) Fr.6–1, (H) Fr.7–1, and (I) Fr.7–2. LC–MS, liquid chromatography‐mass spectrometry.

## Data Availability

The data presented in this study can be found in this published article.

## References

[fsn371092-bib-0001] Bomble, P. , and B. B. Nath . 2022. “Differential Manifestation of RONS and Antioxidant Enzymes in Response to Singular Versus Combinatorial Stress in *Chironomus Ramosus* .” Stress Biology 2: 56. 10.1007/s44154-022-00077-8. 37676561 PMC10442003

[fsn371092-bib-0002] Dong, J. W. , L. Cai , Y. Xing , J. Yu , and Z. T. Ding . 2015. “Re‐Evaluation of ABTS*+ Assay for Total Antioxidant Capacity of Natural Products.” Natural Product Communications 10: 2169–2172. 10.1177/1934578X1501001239. 26882692

[fsn371092-bib-0003] Du, R. , K. Liu , S. Zhao , and F. Chen . 2020. “Changes in Antioxidant Activity of Peptides Identified From Brown Rice Hydrolysates Under Different Conditions and Their Protective Effects Against AAPH‐Induced Oxidative Stress in Human Erythrocytes.” ACS Omega 5: 12751–12759. 10.1021/acsomega.0c00349. 32548459 PMC7288362

[fsn371092-bib-0004] Dubois‐Deruy, E. , V. Peugnet , A. Turkieh , and F. Pinet . 2020. “Oxidative Stress in Cardiovascular Diseases.” Antioxidants 9: 864. 10.3390/antiox9090864. 32937950 PMC7554855

[fsn371092-bib-0005] He, Y. , X. Pan , C. F. Chi , K. L. Sun , and B. Wang . 2019. “Ten New Pentapeptides From Protein Hydrolysate of Miiuy Croaker ( *Miichthys miiuy* ) Muscle: Preparation, Identification, and Antioxidant Activity Evaluation.” LWT 105: 1–8. 10.1016/j.lwt.2019.01.054.

[fsn371092-bib-0006] Hernández‐Ledesma, B. , L. Amigo , I. Recio , and B. Bartolomé . 2007. “ACE‐Inhibitory and Radical‐Scavenging Activity of Peptides Derived From *β*‐Lactoglobulin f(19–25). Interactions With Ascorbic Acid.” Journal of Agricultural and Food Chemistry 55: 3392–3397. 10.1021/jf063427j. 17411066

[fsn371092-bib-0007] Huang, D. , B. Ou , M. Hampsch‐Woodill , J. A. Flanagan , and E. K. Deemer . 2002. “Development and Validation of Oxygen Radical Absorbance Capacity Assay for Lipophilic Antioxidants Using Randomly Methylated *β*‐Cyclodextrin as the Solubility Enhancer.” Journal of Agricultural and Food Chemistry 50: 1815–1821. 10.1021/jf0113732. 11902917

[fsn371092-bib-0008] Jackson, M. J. , and S. O. O'Farrell . 1993. “Free Radicals and Muscle Damage.” British Medical Bulletin 49: 630–641. 10.1093/oxfordjournals.bmb.a072636. 8221028

[fsn371092-bib-0009] Jeong, M. J. , D. S. Lim , S. O. Kim , et al. 2022. “Protection of Oxidative Stress‐Induced DNA Damage and Apoptosis by Rosmarinic Acid in Murine Myoblast C2C12 Cells.” Biotechnology and Bioprocess Engineering 27: 171–182. 10.1007/s12257-021-0248-1.

[fsn371092-bib-0010] Jiang, Y. , X. C. Liu , L. M. Ahrné , and L. H. Skibsted . 2021. “Enthalpy‐Entropy Compensation in Calcium Binding to Acid–Base Forms of Glycine Tyrosine Dipeptides From Hydrolysis of *α*‐Lactalbumin.” Food Research International 149: 110714. 10.1016/j.foodres.2021.110714. 34600648

[fsn371092-bib-0011] Kumar, N. , M. Raghavendra , J. Tokas , and H. R. Singal . 2017. “Milk Proteins: Precursors of Antioxidative Peptides and Their Health Benefits.” In Dairy in Human Health and Disease Across the Lifespan, 313–323. Elsevier. 10.1016/B978-0-12-809868-4.00024-8.

[fsn371092-bib-0012] Lee, D. Y. , Y. S. Chun , J. K. Kim , et al. 2021. “Curcumin Ameliorated Oxidative Stress and Inflammation‐Related Muscle Disorders in c2c12 Myoblast Cells.” Antioxidants 10: 476. 10.3390/antiox10030476. 33802935 PMC8002759

[fsn371092-bib-0013] Lee, J. H. , M. H. Jung , Y. H. Lee , et al. 2013. “Inhibited Apoptosis of C(2)C(1)2 Myoblasts by a *Eupatorium Chinense* Var. *Simplicifolium* Root Extract.” Bioscience, Biotechnology, and Biochemistry 77: 2134–2136. 10.1271/bbb.130333. 24096648

[fsn371092-bib-0014] Lee, M. C. , Y. J. Hsu , C. S. Ho , et al. 2021. “Evaluation of the Efficacy of Supplementation With Planox Lemon Verbena Extract in Improving Oxidative Stress and Muscle Damage: A Randomized Double‐Blind Controlled Trial.” International Journal of Medical Sciences 18: 2641–2652. 10.7150/ijms.60726. 34104096 PMC8176190

[fsn371092-bib-0015] Li, L. , Y. Hu , Y. M. Wang , et al. 2025. “Antioxidant Peptides From the Fruit Source of the Oil Crop *Litsea Cubeba* Ameliorate FFA‐Induced Oxidative Stress Injury: Based on Nrf2/Keap1 Pathway and Molecular Dynamics Simulations.” Food 14: 1707. 10.3390/foods14101707. PMC12111390 40428488

[fsn371092-bib-0016] Li, Y. W. , and B. Li . 2013. “Characterization of Structure‐Antioxidant Activity Relationship of Peptides in Free Radical Systems Using QSAR Models: Key Sequence Positions and Their Amino Acid Properties.” Journal of Theoretical Biology 318: 29–43. 10.1016/j.jtbi.2012.10.029. 23127747

[fsn371092-bib-0017] Nielsen, S. D. H. , N. Liang , H. Rathish , et al. 2024. “Bioactive Milk Peptides: An Updated Comprehensive Overview and Database.” Critical Reviews in Food Science and Nutrition 64: 11510–11529. 10.1080/10408398.2023.2240396. 37504497 PMC10822030

[fsn371092-bib-0018] Nongonierma, A. B. , and R. J. Fitzgerald . 2015. “Milk Proteins as a Source of Tryptophan‐Containing Bioactive Peptides.” Food & Function 6: 2115–2127. 10.1039/c5fo00407a. 26027501

[fsn371092-bib-0019] Ohashi, Y. , R. Onuma , T. Naganuma , et al. 2015. “Antioxidant Properties of Tripeptides Revealed by a Comparison of Six Different Assays.” Food Science and Technology Research 21: 695–704. 10.3136/fstr.21.695.

[fsn371092-bib-0020] Ozturk‐Kerimoglu, B. , A. Heres , L. Mora , and F. Toldrá . 2023. “Antioxidant Peptides Generated From Chicken Feet Protein Hydrolysates.” Journal of the Science of Food and Agriculture 103: 7207–7217. 10.1002/jsfa.12802. 37347843

[fsn371092-bib-0021] Pansarasa, O. , L. Bertorelli , J. Vecchiet , G. Felzani , and F. Marzatico . 1999. “Age‐Dependent Changes of Antioxidant Activities and Markers of Free Radical Damage in Human Skeletal Muscle.” Free Radical Biology and Medicine 27: 617–622. 10.1016/s0891-5849(99)00108-2. 10490283

[fsn371092-bib-0022] Park, C. , H. J. Cha , D. H. Kim , et al. 2023. “Fisetin Protects C2C12 Mouse Myoblasts From Oxidative Stress‐Induced Cytotoxicity Through Regulation of the Nrf2/HO‐1 Signaling.” Journal of Microbiology and Biotechnology 33: 591–599. 10.4014/jmb.2212.12042. 36859395 PMC10236176

[fsn371092-bib-0023] Park, C. , S. Y. Ji , H. Lee , et al. 2021. “Mori Ramulus Suppresses Hydrogen Peroxide‐Induced Oxidative Damage in Murine Myoblast C2C12 Cells Through Activation of AMPK.” International Journal of Molecular Sciences 22: 11729. 10.3390/ijms222111729. 34769159 PMC8583786

[fsn371092-bib-0024] Rival, S. G. , S. Fornaroli , C. G. Boeriu , and H. J. Wichers . 2001. “Caseins and Casein Hydrolysates. 1. Lipoxygenase Inhibitory Properties.” Journal of Agricultural and Food Chemistry 49: 287–294. 10.1021/jf000392t. 11170590

[fsn371092-bib-0025] Rumpf, J. , R. Burger , and M. Schulze . 2023. “Statistical Evaluation of DPPH, ABTS, FRAP, and Folin‐Ciocalteu Assays to Assess the Antioxidant Capacity of Lignins.” International Journal of Biological Macromolecules 233: 123470. 10.1016/j.ijbiomac.2023.123470. 36736974

[fsn371092-bib-0026] Sadat, L. , C. Cakir‐Kiefer , M.‐A. N'Negue , J.‐L. Gaillard , J.‐M. Girardet , and L. Miclo. 2011. “Isolation and Identification of Antioxidative Peptides From Bovine *α*‐Lactalbumin.” International Dairy Journal 21: 214–221.

[fsn371092-bib-0027] Sarmadi, B. H. , and A. Ismail . 2010. “Antioxidative Peptides From Food Proteins: A Review.” Peptides 31: 1949–1956. 10.1016/j.peptides.2010.06.020. 20600423

[fsn371092-bib-0028] Shi, C. , M. Liu , H. Zhao , Z. Lv , L. Liang , and B. Zhang . 2022. “A Novel Insight Into Screening for Antioxidant Peptides From Hazelnut Protein: Based on the Properties of Amino Acid Residues.” Antioxidants 11: 127. 10.3390/antiox11010127. 35052631 PMC8772696

[fsn371092-bib-0029] Shimamura, T. , Y. Sumikura , T. Yamazaki , et al. 2014. “Applicability of the DPPH Assay for Evaluating the Antioxidant Capacity of Food Additives—Inter‐Laboratory Evaluation Study.” Analytical Sciences 30: 717–721. 10.2116/analsci.30.717. 25007929

[fsn371092-bib-0030] Tang, X. , Z. He , Y. Dai , Y. L. Xiong , M. Xie , and J. Chen . 2010. “Peptide Fractionation and Free Radical Scavenging Activity of Zein Hydrolysate.” Journal of Agricultural and Food Chemistry 58: 587–593. 10.1021/jf9028656. 19928919

[fsn371092-bib-0031] Thaipong, K. , U. Boonprakob , K. Crosby , L. Cisneros‐Zevallos , and D. Hawkins Byrne . 2006. “Comparison of ABTS, DPPH, FRAP, and ORAC Assays for Estimating Antioxidant Activity From Guava Fruit Extracts.” Journal of Food Composition and Analysis 19: 669–675. 10.1016/j.jfca.2006.01.003.

[fsn371092-bib-0032] Torkova, A. , O. Koroleva , E. Khrameeva , T. Fedorova , and M. Tsentalovich . 2015. “Structure‐Functional Study of Tyrosine and Methionine Dipeptides: An Approach to Antioxidant Activity Prediction.” International Journal of Molecular Sciences 16: 25353–25376. 10.3390/ijms161025353. 26512651 PMC4632805

[fsn371092-bib-0033] Wang, J. , J. Liu , A. John , et al. 2022. “Structure Identification of Walnut Peptides and Evaluation of Cellular Antioxidant Activity.” Food Chemistry 388: 132943. 10.1016/j.foodchem.2022.132943. 35436638

[fsn371092-bib-0034] Wang, X. , L. Lei , and J. Zhang . 2012. “Antioxidant and Anti‐Fatigue Activities of Flavonoids From Puerariae Radix.” African Journal of Traditional, Complementary, and Alternative Medicines 9: 221–227. 10.4314/ajtcam.v9i2.6. PMC3746631 23983338

[fsn371092-bib-0035] Wang, Y. M. , M. X. Ge , S. Z. Ran , X. Pan , C. F. Chi , and B. Wang . 2025. “Antioxidant Peptides From Miiuy Croaker Swim Bladders: Ameliorating Effect and Mechanism in NAFLD Cell Model Through Regulation of Hypolipidemic and Antioxidant Capacity.” Marine Drugs 23: 63. 10.3390/md23020063. 39997187 PMC11857530

[fsn371092-bib-0036] Wang, Y. M. , X. Y. Li , J. Wang , Y. He , C. F. Chi , and B. Wang . 2022. “Antioxidant Peptides From Protein Hydrolysate of Skipjack Tuna Milt: Purification, Identification, and Cytoprotection on H_2_O_2_ Damaged Human Umbilical Vein Endothelial Cells.” Process Biochemistry 113: 258–269. 10.1016/j.procbio.2022.01.008.

[fsn371092-bib-0037] Watanabe, J. , T. Oki , J. Takebayashi , et al. 2012. “Method Validation by Interlaboratory Studies of Improved Hydrophilic Oxygen Radical Absorbance Capacity Methods for the Determination of Antioxidant Capacities of Antioxidant Solutions and Food Extracts.” Analytical Sciences: The International Journal of the Japan Society for Analytical Chemistry 28: 159–165. 10.2116/analsci.28.159. 22322809

[fsn371092-bib-0038] Wu, C. , J. Guo , H. Jian , et al. 2023. “Bioactive Dipeptides Enhance the Tolerance of Lager Yeast to Ethanol‐Oxidation Cross‐Stress by Regulating the Multilevel Defense System.” Food Microbiology 114: 104288. 10.1016/j.fm.2023.104288. 37290871

[fsn371092-bib-0039] Wu, C. , C. Wang , J. Guo , et al. 2024. “Plant‐Derived Antioxidant Dipeptides Provide Lager Yeast With Osmotic Stress Tolerance for Very High Gravity Fermentation.” Food Microbiology 117: 104396. 10.1016/j.fm.2023.104396. 37919005

[fsn371092-bib-0040] Xie, N. , C. Wang , J. Ao , and B. Li . 2013. “Non‐Gastrointestinal‐Hydrolysis Enhances Bioavailability and Antioxidant Efficacy of Casein as Compared With Its in Vitro Gastrointestinal Digest.” Food Research International 51: 114–122. 10.1016/j.foodres.2012.12.001.

[fsn371092-bib-0041] Yaffe, D. , and O. Saxel . 1977. “Serial Passaging and Differentiation of Myogenic Cells Isolated From Dystrophic Mouse Muscle.” Nature 270: 725–727. 10.1038/270725a0. 563524

[fsn371092-bib-0042] Zhang, H. , Y. Luo , and H. Liu . 2020. “Walnut protein peptide with antioxidant and DPP‐IV (dipeptidyl peptidase‐IV) inhibiting functions. Chinese Patent CN107828842B, 2020.05.22.”

[fsn371092-bib-0043] Zhang, Y. , Y. Li , Z. Quan , P. Xiao , and J. A. Duan . 2024. “New Insights Into Antioxidant Peptides: An Overview of Efficient Screening, Evaluation Models, Molecular Mechanisms, and Applications.” Antioxidants 13: 203. 10.3390/antiox13020203. 38397801 PMC10886007

[fsn371092-bib-0044] Zheng, L. , L. Lin , G. Su , Q. Zhao , and M. Zhao . 2015. “Pitfalls of Using 1,1‐Diphenyl‐2‐Picrylhydrazyl (DPPH) Assay to Assess the Radical Scavenging Activity of Peptides: Its Susceptibility to Interference and Low Reactivity Towards Peptides.” Food Research International 76: 359–365. 10.1016/j.foodres.2015.06.045. 28455015

[fsn371092-bib-0045] Zhu, L. , H. Xiong , X. Huang , et al. 2022. “Identification and Molecular Mechanisms of Novel Antioxidant Peptides From Two Sources of Eggshell Membrane Hydrolysates Showing Cytoprotection Against Oxidative Stress: A Combined in Silico and in Vitro Study.” Food Research International 157: 111266. 10.1016/j.foodres.2022.111266. 35761579

[fsn371092-bib-0046] Zhu, X. , D. Sun‐Waterhouse , Q. Tao , W. Li , D. Shu , and C. Cui . 2020. “The Enhanced Serotonin (5‐HT) Synthesis and Anti‐Oxidative Roles of Trp Oligopeptide in Combating Anxious Depression C57BL/6 Mice.” Journal of Functional Foods 67: 103859. 10.1016/j.jff.2020.103859.

