# Supplementary material for: Antioxidant Properties of Whey Protein‐Derived Peptides: Radical Scavenging and Cytoprotective Effects
Source: Food Sci Nutr. 2025 Oct 17;13(10):e71092. doi: 10.1002/fsn3.71092 (PMC12534199; doi:10.1002/fsn3.71092)
Supplement: Supplementary file 1 — Table S1: Amino acid composition of the whey protein hydrolysate. Table S2: Fractionation conditions for each fraction. Figure S1: HPLC chromatogram and H‐ORAC activity of each fraction. Frs.1–7, which had high H‐ORAC activity among the 192 fractions of whey protein hydrolysate, were fractionated again, and H‐ORAC activity was measured. The HPLC chromatogram is shown in black, and the H‐ORAC values of the fractions are indicated in red. Arrows indicate fractions with high H‐ORAC activity. Graphs show: (A) Fr.1, (B) Fr.2, (C) Fr.3, (D) Fr.4, (E) Fr.5, (F) Fr.6, and (G) Fr.7. H‐ORAC; hydrogen‐oxygen radical absorbance capacity; HPLC, high‐performance liquid chromatography; TE, Trolox equivalent. Figure S2: Dominant fragment ions of each fraction. Each fraction was refractionated and subjected to LC–MS/MS to determine the fraction with the highest hydrogen‐oxygen radical absorbance capacity activity. The figure shows the dominant fragments in LC–MS. The vertical axis presents the relative abundance, and the horizontal axis presents the m/z values. The respective graphs show: (A) Fr.1–1, (B) Fr.1–2, (C) Fr.2–1, (D) Fr.3–1, (E) Fr.4–1, (F) Fr.5–1, (G) Fr.6–1, (H) Fr.7–1, and (I) Fr.7–2. LC–MS, liquid chromatography‐mass spectrometry. [file FSN3-13-e71092-s001.docx]

Supplementary Table 1. Amino acid composition of the whey protein hydrolysate.

| Amino acid | Concentration [mg/WPH 100 g] |
| --- | --- |
| Ala | 4280 |
| Arg | 1970 |
| Asx ^*1^ | 9200 |
| Cys ^*2^ | 1750 |
| Glx ^*3^ | 15500 |
| Gly | 1480 |
| His | 1490 |
| Ile | 5290 |
| Leu | 8100 |
| Lys | 7620 |
| Met ^*4^ | 1530 |
| Phe | 2350 |
| Pro | 5260 |
| Ser | 4320 |
| Thr | 6110 |
| Trp | 1230 |
| Tyr | 2350 |
| Val | 4670 |

*1 Total asparagine and aspartic acid

*2 Detected as cystine

*3 Total glutamine and glutamic acid

*4 Detected as methionine sulfone

Supplementary Table 2. Fractionation conditions for each fraction.

| **Fraction No.** | **Gradient conditions** |
| --- | --- |
| Fr.1  Fr.2  Fr.3  Fr.4 | ＜0–5 min＞2％　＜5–41 min＞2-35%　＜41–45 min＞35–80％　 ＜45–50 min＞80％　＜50–55 min＞2％ |
| Fr.5 | ＜0–1 min＞2％　＜1–3 min＞2-10％　＜3–7 min＞10％　 ＜7–40 min＞10-40%　＜40–45 min＞40–80％　＜45–50 min＞80％　 ＜50–55 min＞2％ |
| Fr.6  Fr.7 | ＜0–1 min＞2％　＜1–3 min＞2-15％　＜3–7 min＞15％　 ＜7–40 min＞15-45%　＜40–45 min＞45-80％　＜45–50 min＞80％　 ＜50–55 min＞2％ |

(A)


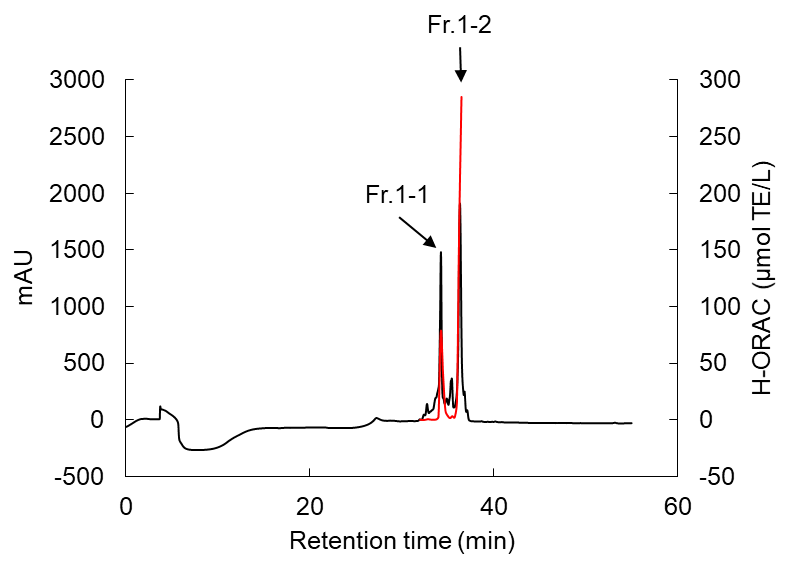


(B)


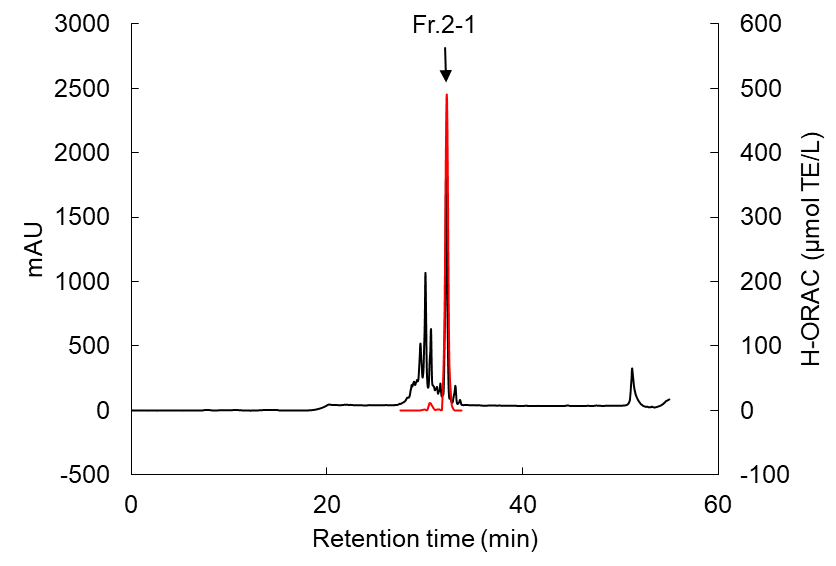


(C)


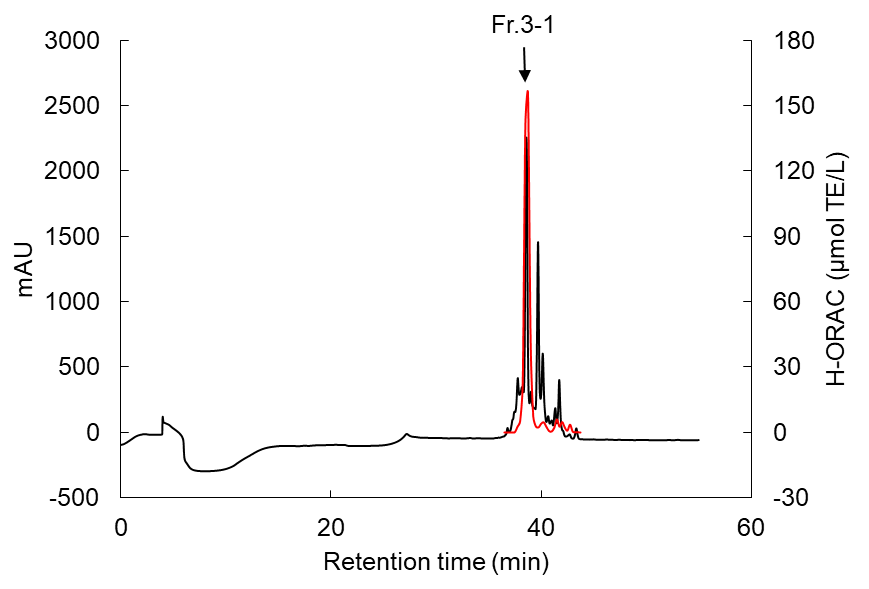


(D)


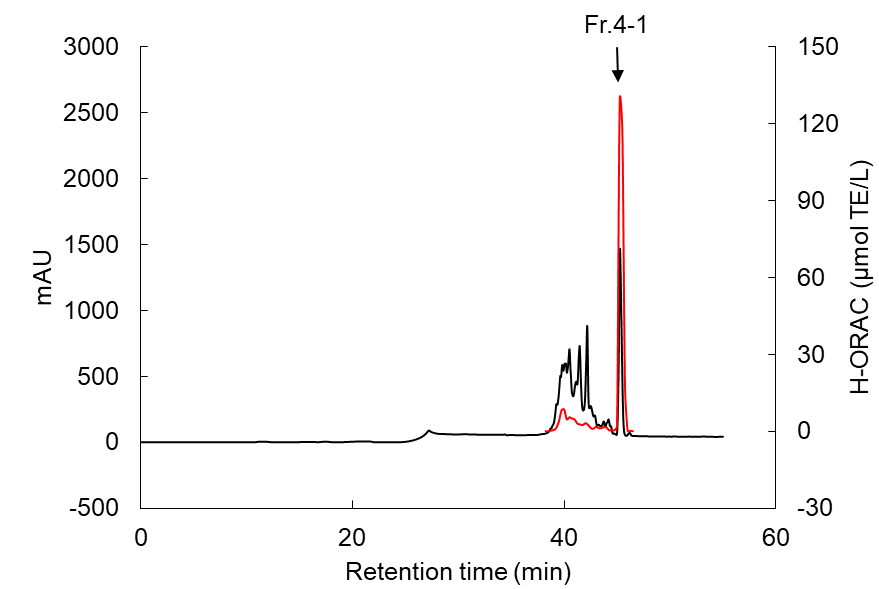


(E)


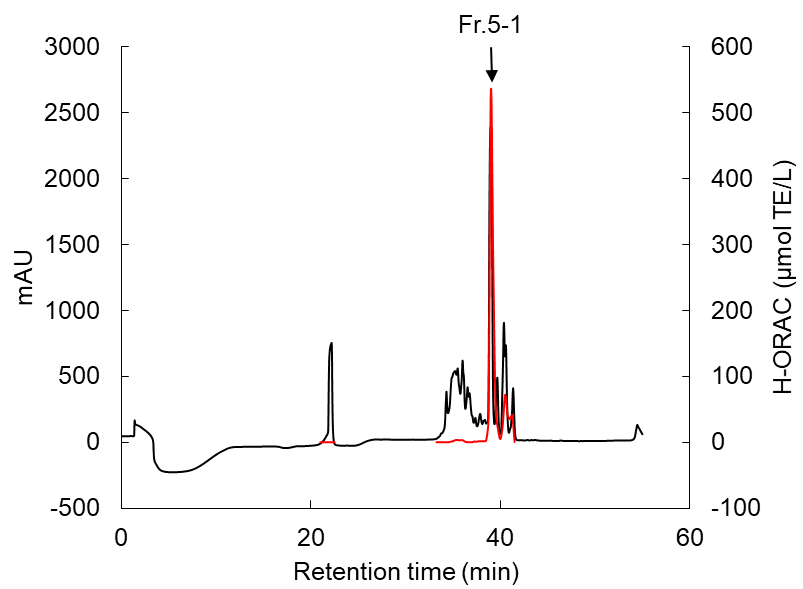


(F)


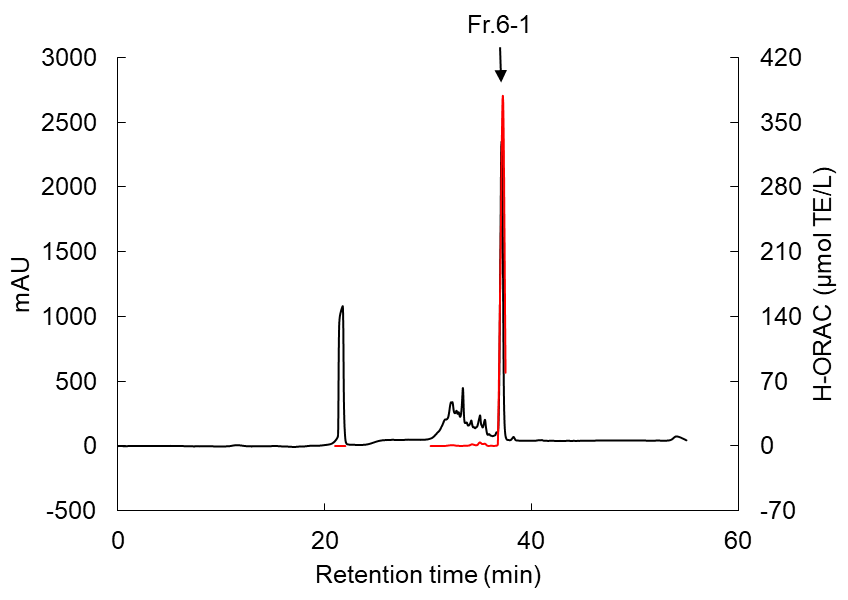


(G)


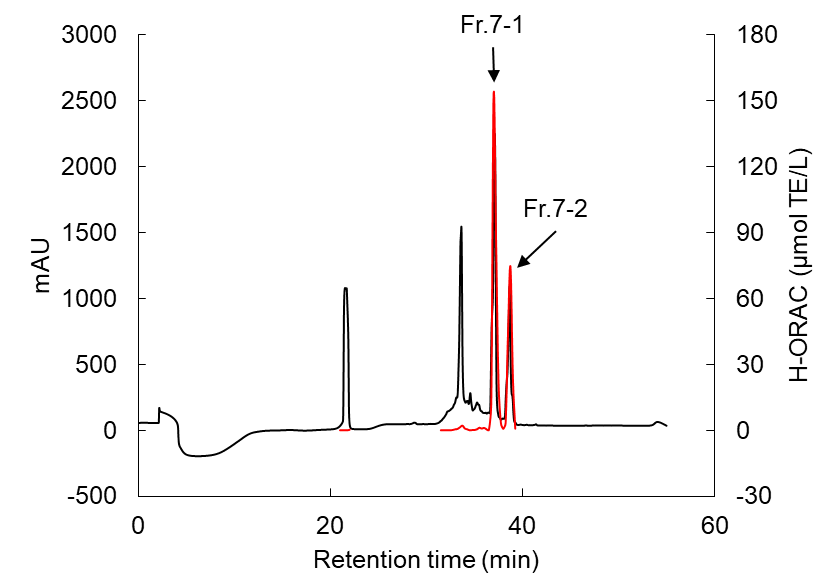


Supplementary Figure 1. HPLC chromatogram and H-ORAC activity of each fraction. Frs.1–7, which had high H-ORAC activity among the 192 fractions of whey protein hydrolysate, were fractionated again, and H-ORAC activity was measured. The HPLC chromatogram is shown in black and the H-ORAC values of the fractions are indicated in red. Arrows indicate fractions with high H-ORAC activity. Graphs show: (A) Fr.1, (B) Fr.2, (C) Fr.3, (D) Fr.4, (E) Fr.5, (F) Fr.6, and (G) Fr.7. H-ORAC; hydrogen-oxygen radical absorbance capacity; HPLC, high-performance liquid chromatography; TE, Trolox equivalent.

(A)


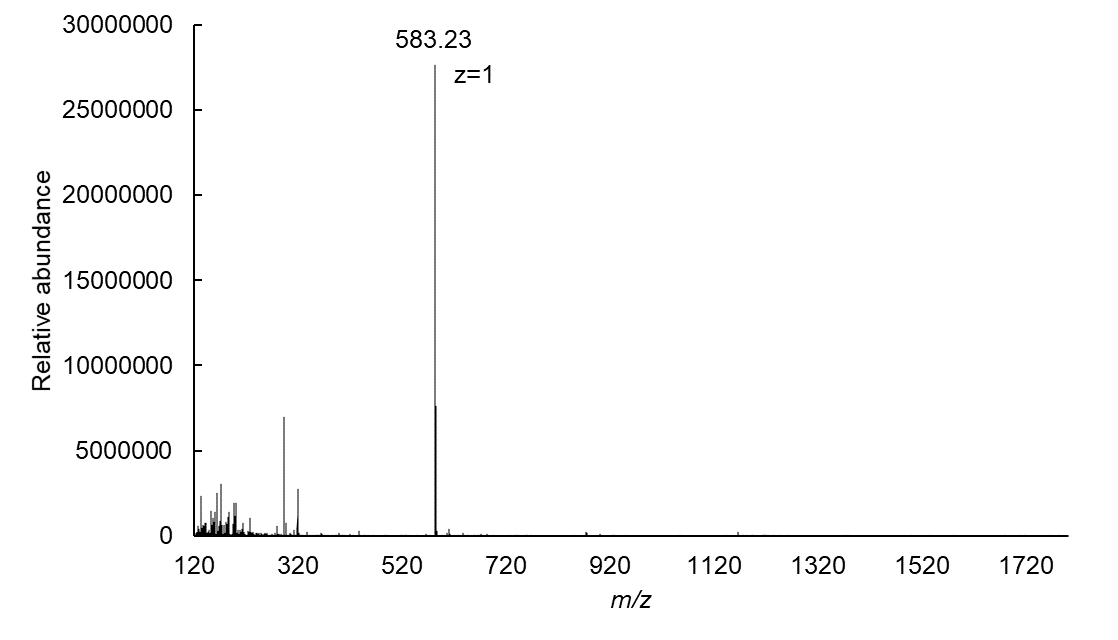


(B)


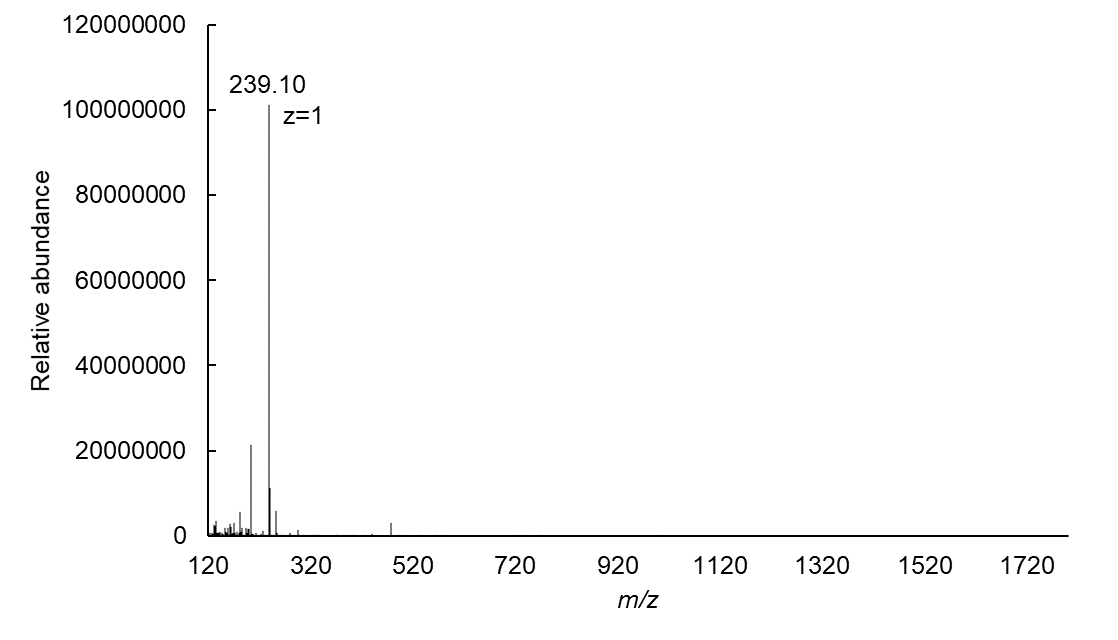


(C)


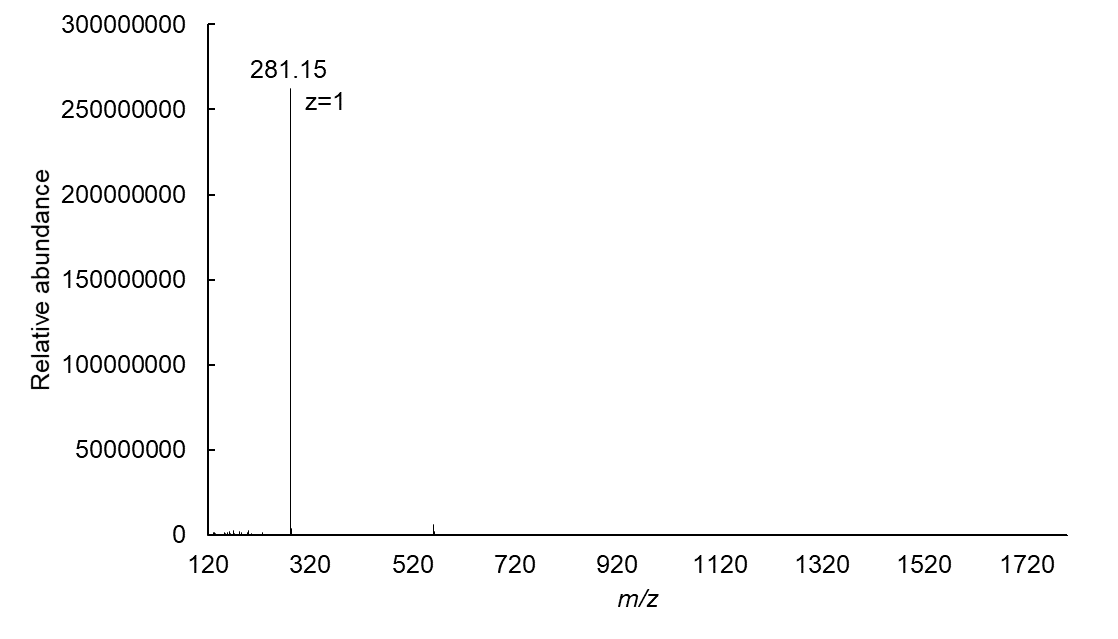


(D)


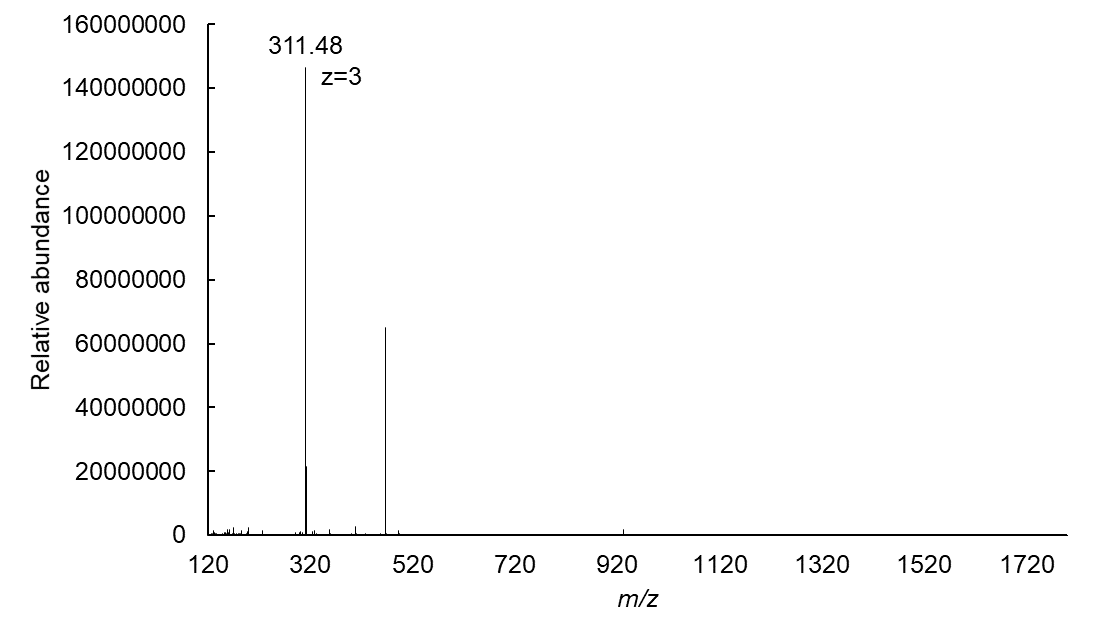


(E)


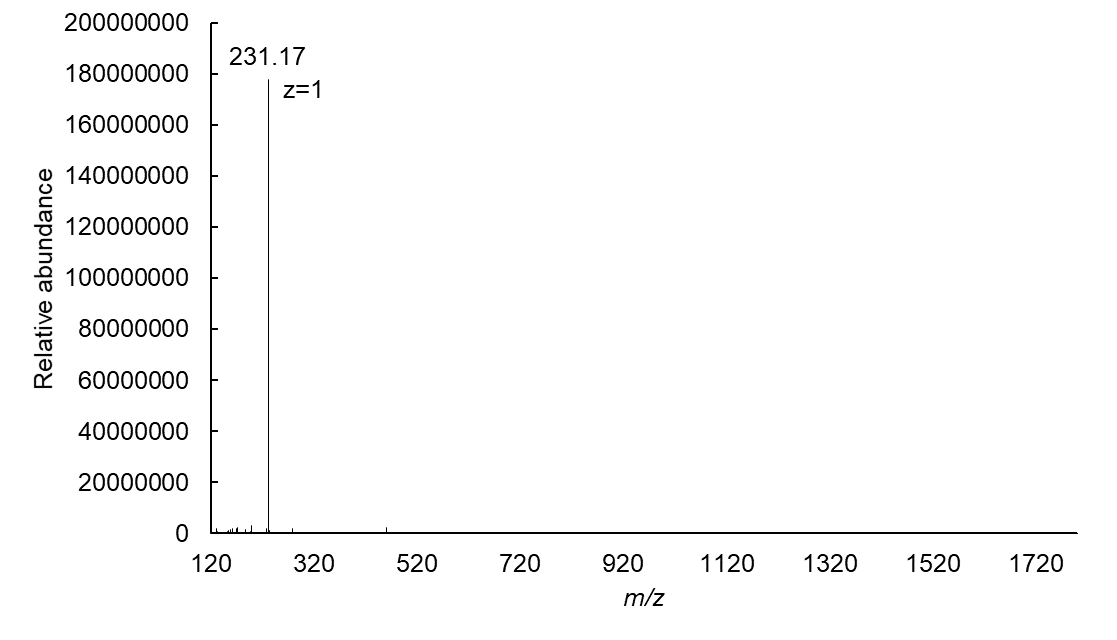


(F)


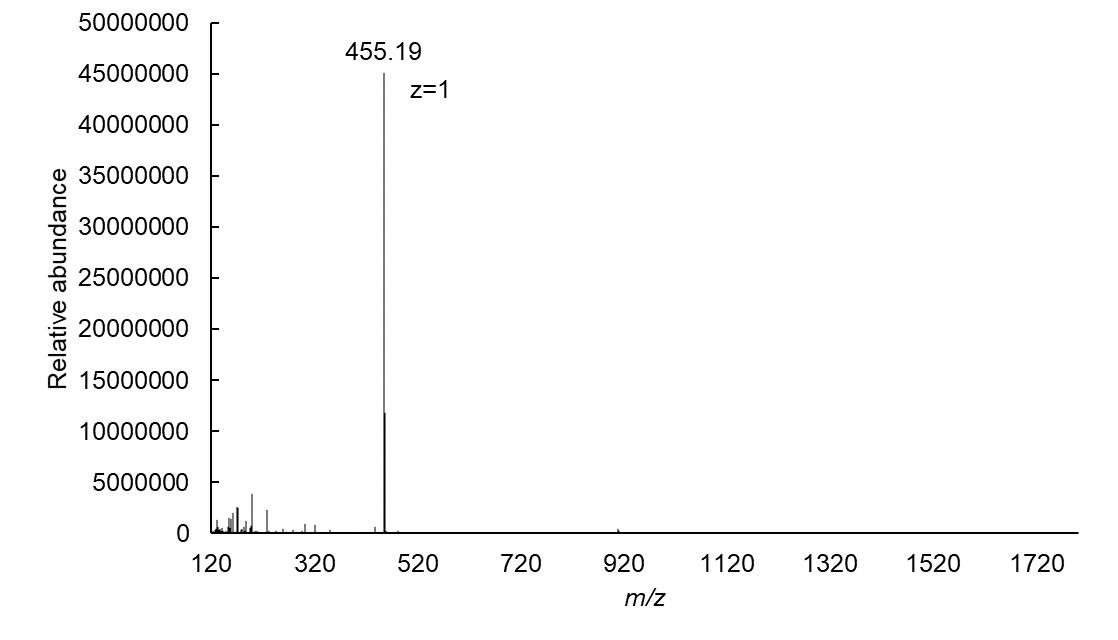


(G)


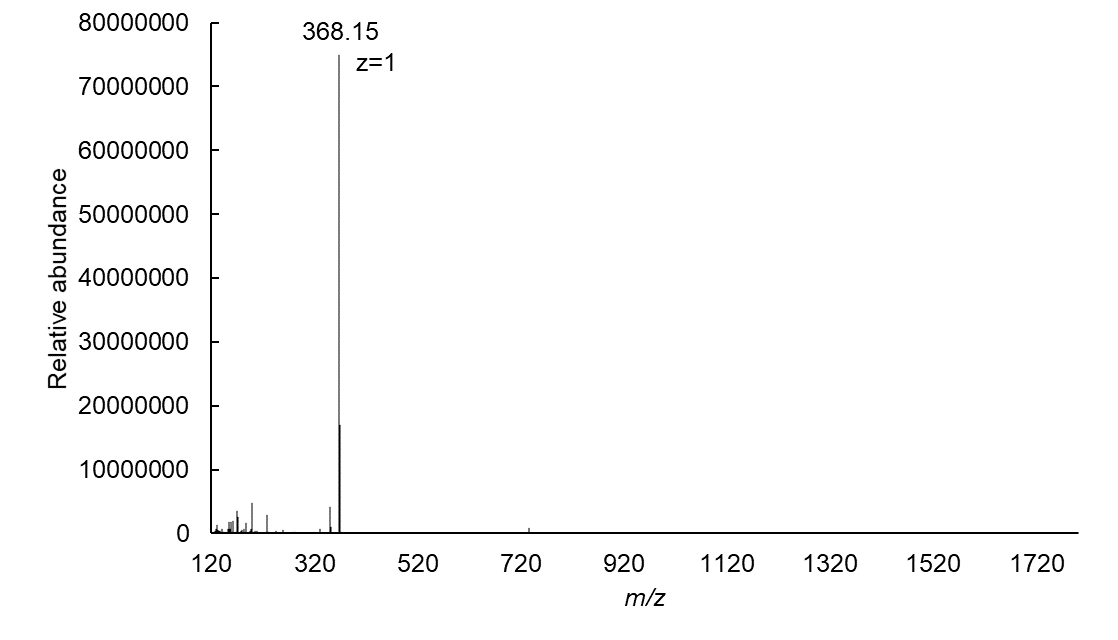


(H)


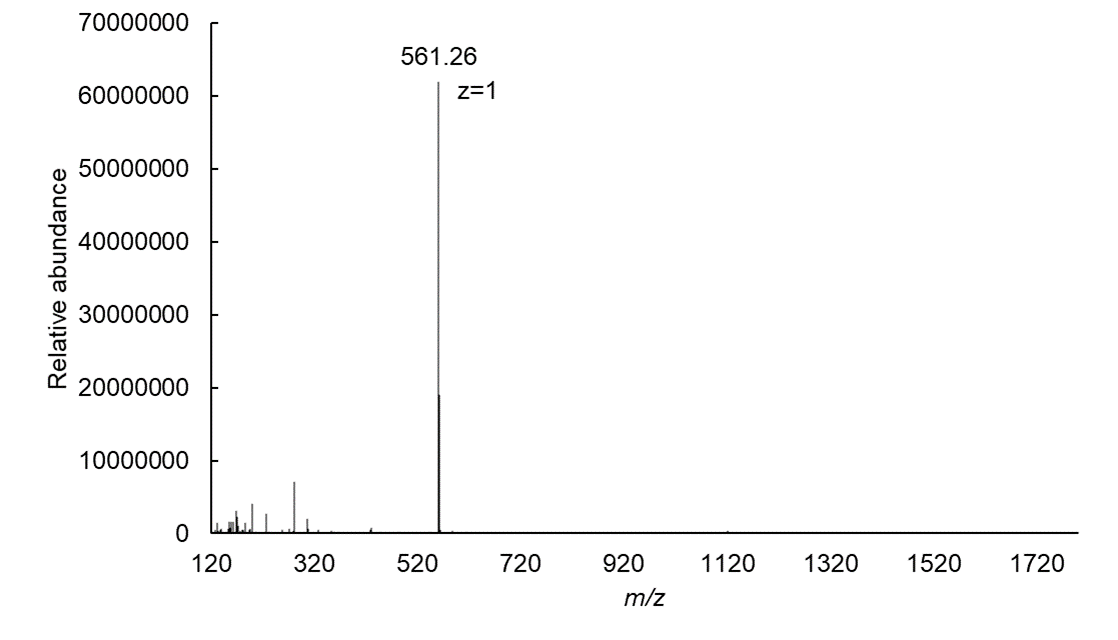


(I)


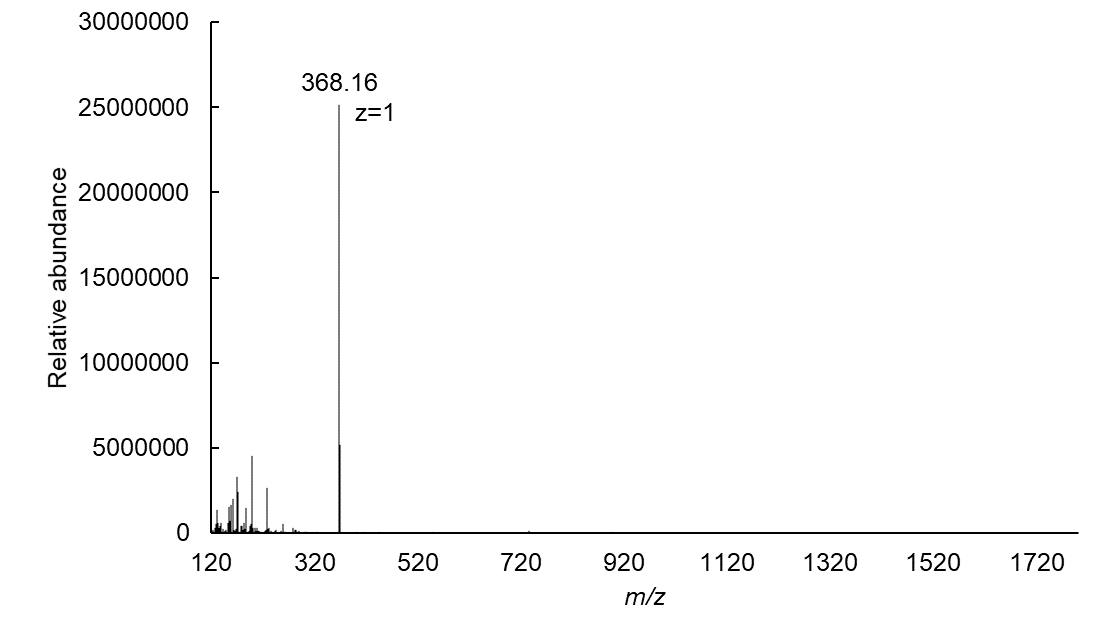


Supplementary Figure 2. Dominant fragment ions of each fraction. Each fraction was refractionated and subjected to LC-MS/MS to determine the fraction with the highest hydrogen-oxygen radical absorbance capacity activity. The figure shows the dominant fragments in LC-MS. The vertical axis presents the relative abundance, and the horizontal axis presents the *m/z* values. The respective graphs show: (A) Fr.1-1, (B) Fr.1-2, (C) Fr.2-1, (D) Fr.3-1, (E) Fr.4-1, (F) Fr.5-1, (G) Fr.6-1, (H) Fr.7-1, and (I) Fr.7-2. LC-MS, liquid chromatography-mass spectrometry.
